# Older people and their families’ perceptions about their experiences with interprofessional teams

**DOI:** 10.1002/nop2.123

**Published:** 2018-02-07

**Authors:** Sherry Dahlke, Kim Steil, Rosalie Freund‐Heritage, Marnie Colborne, Susan Labonte, Adrian Wagg

**Affiliations:** ^1^ Faculty of Nursing University of Alberta Edmonton AB Canada; ^2^ Glenrose Rehabilitation Hospital Edmonton AB Canada; ^3^ Alberta Health Services Edmonton AB Canada; ^4^ Department of Medicine University of Alberta Edmonton AB Canada

**Keywords:** communication, family involvement in care, interprofessional teams, older adults

## Abstract

**Aim:**

To examine older people and their families’ perceptions about their experiences with interprofessional teams.

**Design:**

Naturalistic inquiry using qualitative descriptive methods to provide a comprehensive summary of older people and their families’ experiences with interprofessional teams.

**Methods:**

Interviews were conducted with 22 people from 11 families. The families had experiences with teams in a variety of settings, such as community, residential care and hospital. Data were analysed using inductive content analysis. NiVivo was used to record preliminary codes. Analysis included comparing and contrasting families’ experiences.

**Results:**

Older people and their families wanted communication about what was going on, regardless of whether the news was good, bad or unknown. They also wanted care that took the concerns of the older person into consideration. Communication was a necessary ingredient to ensuring that the older person's unique concerns were known to the interprofessional team. These percepectives were discussed in the themes of communication and patient‐centred care.

## 1 INTRODUCTION

**Figure 1:**
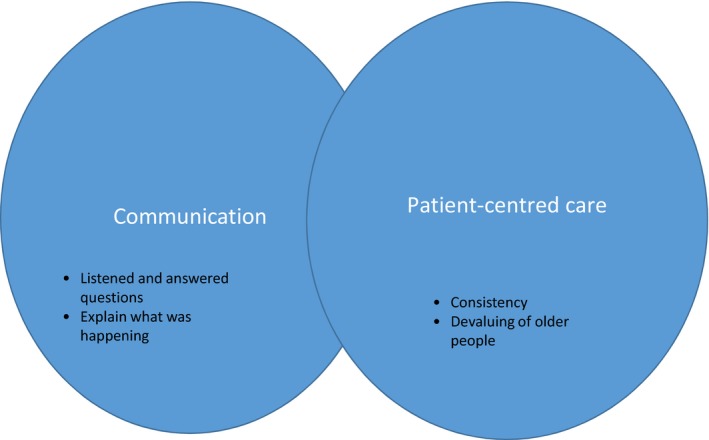
Perceptions about experiences with interprofessional teams

The health care of older persons is often complex due to an increased incidence of long‐term illnesses, atypical presentations of acute illness and changing social circumstances (Arbaji et al., 2010; Fedarko, 2011; Hartgerink et al., 2014) . This complexity increases the likelihood of multiple professional disciplines becoming involved in the management of older people's health (Smith‐Carrier & Neysmith, 2014; World Health Organization [WHO], 2016). When multiple disciplines are involved they often work in teams known by a variety of names, such as multidisciplinary, interdisciplinary, transdisciplinary and interprofessional; in this study, we use the term interprofessional team, defined as two or more professional disciplines in communication with one another about older people's health (Fox & Reeves, 2015). Researchers have reported some successes in improving the care of older people when interprofessional teams are involved, (Arbaji et al., 2010; Barrow, McKimm, Gasquoine, & Rowe, 2014, Boult et al., 2009; WHO, 2016). However, the processes teams use to incorporate the views of older people and their families in their decision‐making, is unclear. Older people may be more dependent on caregivers, such as friends or family, and the more people involved the more the potential for miscommunication. To understand better how to avoid miscommunication, it is important to understand older people and their families’ perspectives on their experiences with interprofessional teams Figure 1.

## 2 BACKGROUND

Interprofessional teams are frequently promoted as a means to provide quality, safety and efficiency of care within health care (Health Canada, 2007; Reeves, Lewin, Espin, & Zwarenstein, 2010; WHO, 2010). As well as interprofessional team involvement, actively involving older people in their own care has been associated with improved health and more effective healthcare utilization (Berglund et al., 2013; Hochhalter, Song, Rush, Sklar, & Stevens, 2010). However, older people face obstacles to active engagement in their care, such as low health literacy (Wolf, Gazmararian, & Baker, 2005) or delirium (Holroyd‐Leduc, Khandwala, & Sink, 2010). Moreover, there is an increased incidence of dementia with advancing age which can compromise individuals’ ability to interact effectively with multiple professionals (WHO, 2015). In cases of dementia, delirium or both, the families or informal caregivers of older people are frequently involved in decision‐making on their behalf (Legare et al., 2014). In this situation, family involvement adds to the complexity of care and must be considered by interprofessional teams working in this area.

Scholars who have examined the characteristics of effective interprofessional teamwork suggest that to be effective, team members must have social competence, a willingness to share information, to be able to negotiate and solve problems, (Mickan & Rodger, 2005). Manser's (2009) review of the literature about patient safety and teamwork indicated that teams require patterns of communication, coordination and leadership to support their effectiveness. Yet, there is evidence that power issues, confusion about roles, inconsistent use of language and inadequate organizational supports are common challenges for interprofessional teams (Barrow et al., 2014; Finn, Learmonth, & Reedy, 2010; Fox & Reeves, 2015). These challenges reflect social, political and economic complexities associated with interprofessional collaboration (Essen, Freshwater, & Cahill, 2015; Fox & Reeves, 2015). Scholars have developed an interprofessional framework identifying the complexities to teamwork as: relational (personalities and social interactions); contextual (culture, gender, and economics); organizational challenges; and process issues, such as routines and rituals, complexity, urgency and task shifting (Reeves et al., 2010). Unfortunately, this framework fails to include the perspectives of either care recipients or their families.

All of the challenges and complexities of interprofessional teamwork influence how professionals communicate (Reeves, 2012; Rowlands & Callen, 2013). Thus, although effective interprofessional collaboration is believed to reduce duplication, clinical errors and enhance the quality of care (Morey et al., 2002; Schmitt, 2001, 2006), beliefs about the efficacy of interprofessional teams are shrouded in a lack of understanding about the processes (the how) by which professionals collaborate and communicate (Brandt, Lutfiyya, King, & Chioreso, 2014; Jones & Jones, 2011; Lemieux‐Charles & McGuire, 2006; Paradis et al., 2014; Reeves et al., 2010). In other words, despite three decades of literature examining the efficacy of interprofessional teams, there remains an absence of evidence to guide teams in patterns of communication that will support collaboration with older people and their families.

There is also little research examining how interprofessional teams work with older people and their families. In a recent study, a scoping review which examined the factors associated with interprofessional teams’ success when working with cognitively impaired older people, only 3 of 34 papers reviewed reported any information‐ how‐be‐it‐ scant‐ about how team members worked with older persons or their families (Dahlke et al., 2017). This suggests that either researchers did not report this information, or that teams did not actively engage with this population. We found only four other studies that examined older people's experiences with interprofessional teams (Berglund et al., 2013; Eloranta, Routasalo, & Arve, 2008; Hochhalter et al., 2010; Lamb et al., 2014). Berglund et al. (2013) suggested that older people were satisfied with their care because they got attention and their needs were met when the interprofessional team was involved. Older people who participated in this study were cognitively intact and thus more able to advocate for themselves and to interact with interprofessional teams than would those with cognitive impairment. Lamb and colleagues reported that cancer care recipients (not all were older people) wanted to make decisions about their care with the help of families and healthcare professionals. The perspectives of older people and in particular those who may be less able to communicate their desires and needs were missing from this study. Eloranta et al. (2008) suggested that interprofessional collaboration could improve the care of community dwelling older people. However, older people in this study did not recognize that collaboration was occurring, leaving questions about what type of interaction older people wanted and would find helpful. In a workshop with older people aimed at improving their engagement with interprofessional teams, older people reported improved self‐efficacy but did not increase their engagement (Hochhalter et al., 2010), leaving questions about what type of communication older people want.

Taken together, little is known about how (the processes) interprofessional teams communicate and work with older people and their families. Moreover, little is known about older people and their families’ perspectives on their experiences with interprofessional teams. Learning how older people and their families’ view these interactions is a first step in understanding what they want from interprofessional teams. Therefore, the aim of this exploratory study was to gain an understanding of these perspectives about experiences with interprofessional healthcare teams.

## 3 METHODS

### 3.1 Design

This was a naturalistic inquiry using qualitative descriptive methods to provide a comprehensive summary of older people and their families’ perspectives on their experiences with interprofessional teams (Sandelowski, 2000, 2010). Data were collected from June to December 2015 and included individual, dyad and triad interviews.

### 3.2 Sampling

We used purposeful sampling to include older people and/or his or her family who had experiences with an interprofessional team. The clinicians from three interprofessional teams (an acute care team, a community team and a rehabilitation team) provided older people and their families with an information letter about the study that included the purpose of the study, information on potential risks, and the researchers’ contact information. Older people and/or family members who were interested in participating contacted the researcher or consented to have the clinician share their contact information with the researcher.

Whenever possible we interviewed the older person and their family member together. Unfortunately, this was not always possible either due to family members availability, or because the older person with dementia was unable to remember or describe their experiences

### 3.3 Ethical considerations

Research Ethics Committee approval was obtained from the University of record and operational approval from the participating health authority. All families were informed about the study, the voluntary nature of participation and confidentiality. All signed consent forms prior to their participation. Consent was obtained from older people when family and healthcare professionals agreed that they could provide informed consent. When the older person was unable to provide informed consent, their family provided consent and the older person received a simple explanation of the study and was asked for their assent prior to data collection.

### 3.4 Data collection

Interviews occurred at a place of participants’ choosing. Most of the interviews were conducted in participants’ homes. Two were conducted in a quiet corner of a coffee shop at a hospital. The first author conducted all of the interviews using a semi‐structured interview guide after obtaining informed consent. Questions were open‐ended, focusing on exploring participants’ experiences and perspectives. Questions included but were not limited to: “tell me about your (or your family member's) experiences being cared for by a variety of healthcare professionals”; “How were your perspectives considered by the professionals?”; “How were your family's perspectives included?”; and “How would you like to engage with interprofessional teams?” The last question was included due to Eloranta et al. (2008) finding that despite professionals belief that they were collaborating with older people, the older people did not recognize the engagement as collaboration, leaving questions about what type of engagement older people want with interprofessional teams.

### 3.5 Data analysis

Data were audio‐recorded and then transcribed verbatim; all identifiers were removed prior to data analysis. When interviews provided no new perspectives, data were considered saturated. Inductive content analysis was used to analyse the data (Hsieh & Shannon, 2005; Sandelowski, 2000). The aim of content analysis is to interpret participants’ perspectives as close to the data as possible, avoiding the use of preconceived codes or categories (Hsieh & Shannon, 2005). Analysis began with two of the researchers (S.D. & M.C) reading the transcripts carefully line‐by‐line, highlighting text that described older people and their families’ perspectives. Key words or codes were entered into NiVivo as preliminary codes. Next, the first author and two others (M.C. & S.L.) independently analysed the data using line‐by‐line coding and compared their codes to the NiVivo codes. After discussion on the codes, S.D., M.C. and S.L reached an agreement about codes that represented the data. They then grouped similar codes together to form categories. The categories were then examined for similarities and differences to develop meaningful themes, using quotes from participants to support themes and categories. Discussion on analysis of the data also included comparing and contrasting older people and their families’ experiences. The first author then wrote a draft of the findings based on the analysis and discussions that had occurred among S.D., M.C. and S.L. Finally, an iterative analytic process among all authors occurred in which categories and themes were interrogated for whether or not they were a realistic representation of the data. This process served to further develop the themes as a description of older people and their families’ perspectives about engaging with interprofessional teams.

### 3.6 Validity and reliability

Analytical rigour was assured through incorporating general considerations for qualitative research. The trustworthiness of this study was enhanced by attending to the characteristics described by Grove, Gray, and Burns (2015). The credibility of the findings was enriched by triangulation of the data among the researchers. Moreover, that data for each of the themes presented both positive and negative examples to demonstrate the range of older people and their families’ experiences also enhances credibility. The attention to describing the contexts and the rich description provided by participants’ quotes supported the categories and themes and enhanced the transferability of these findings. A transparent analytical decision trail, as described in the analysis section, and the rigorous discussions among the co‐authors contribute to the dependability of the findings. Discussing preliminary codes, use of NVivo and the rigorous conversations among the co‐authors about whether the categories and themes were a realistic representation of the data contributed to the dependability and confirmability of the findings.

## 4 FINDINGS

Eleven families (22 individuals) participated in this study. Table 1 contains information about the family groupings and their ages. Four group and nine individual interviews were conducted. Older people and their spouses ranged in age from 65 to 89 years and adult children ranged in age from 47 to 66 years. Experiences with interprofessional teams were described from an older person's perspective (one), a family perspective that included the older person (three) and a family perspective in which the older person's perspective was not included due to advanced dementia (seven). Several families talked about their experiences with interprofessional teams in more than one setting; these settings included community, acute care, rehabilitation and nursing homes. Older people and their families agreed that families should be included in communication and healthcare decision‐making. This is due to the belief “that [families] really know best the parent's needs, or the safest and their best interest” (Carol, daughter). Older people and their families wanted communication about what was going on, regardless of whether the news was good, bad or unknown. They also wanted care that took the unique concerns of the older person into consideration. Communication was a necessary ingredient to ensuring that the older person's concerns were known to the interprofessional team. These perspectives are discussed further in the themes of communication and patient‐centred care.

**Table 1:** Family structure

Number	Family members	Ages
1	Mable (daughter)	55
2	Lucy (wife)	81
	Zack	86
3	Louise (wife)	65
	Bob	79
4	Martha	89
	Ray (son)	66
	Carol (daughter)	61
	Pam (daughter)	54
5	Alice (daughter)	73
6	Grace (wife)	80
	Ed	83
7	Irwin	79
	Gladys (wife)	76
	Brent (son)	47
8	Marliss (daughter)	57
	Jenny	79
9	Deloris	80
	Earl (husband)	85
10	Sandy (daughter)	52
	Albert	85
11	Alice	78

### 4.1 Communication

Older people and their families identified effective communication both within the team and to them as necessary ingredients in ensuring that the older person received care that took their needs into consideration. Older people and their families felt confident that care would meet the older person's needs when professionals *listened and answered questions*, and *explained what was happening*.

#### 4.1.1 Listened and answered questions

An important element of good communication was active listening to older people and their families’ concerns. Active listening required professionals to engage in conversation with the older person and their family until it was crystal clear that both the professional and the older person/family understood the message[s] being conveyed. When asked what professionals should do, one older person explained: “listen to them. And make sure they understand you” (Alice, older person). When it worked well, explanations were worded in ways that made it easy for older people and their families to understand. One older person remarked: “I could ask them questions and understood what they were saying” (Alice, older person). Another husband explained: “They keep you well informed. You stop asking questions only when you run out of questions” (Earl, husband). This husband explained that he was impressed by the interprofessional team's willingness to answer his and his children's, questions about their mother.

Listening and answering the questions of families as well as the older person was important because if the family understood what was happening they would be able to share the older person's values when the older person was unable to do so. This allowed families to contribute to the plan of care. One older person explained how having family understand the plan of care was reassuring because “the family can explain to [the older person] what they're trying to do” (Martha, older person). Family members who understood the plan of care and were trusted by the older person were able to help explain the care plan in a way that could be more easily understood by the care recipient.

#### 4.1.2 Explain what was happening

Older people and their families wanted information about what was going on and the plan of care. When professionals took the time to explain what was happening, families felt recognition of the family unit and felt confident that their family members’ needs were being considered. As one daughter relayed: “they explained how the whole process would probably play out. I didn't worry about her at all. I really felt like they cared about me too” (Mabel, daughter). As this daughter explained open communication contributed to confidence that the team was interested in their older family member's needs.

Some of the teams identified a specific member from the interprofessional team that families could go to for questions about how the older person was progressing. This person could then help to direct them to the professional who could answer specific questions. As one daughter explained: “The social worker is the contact person that we can talk to. The physio, the occupational therapist, they keep in contact with us too. The nurses give you information; it is really well done” (Sandy, daughter). Having one person to contact provided direction in the sometimes confusing healthcare environment. Moreover, when the interprofessional team made it a practice to explain what was going on, older people and their families were reassured by the culture of open communication.

Just as family were reassured when they received communication about the older person, if communication was ineffective among the interprofessional team members or between the healthcare providers and the older person/family, the result was family/patient anxiety and eventually suboptimal care. As an example—Bob had dementia and was not able to provide answers to many of the healthcare providers’ questions. As a result, his wife Louise stayed close to his side. She explains their experience:

“There was a total breakdown [of] communication, everything fell apart. They totally lost track of what was going on, why he was there, his history. He didn't know who his nurse was… with dementia, you've got to introduce yourself more than once and that didn't happen. His food would sit there and nobody would help him. I felt like there wasn't a team. Now I know there was, because people were coming and going all the time but I don't know who they were. I don't know what their purpose was [or] what they were trying to accomplish with him. People would just come in and they'd start doing things and talking to him. I was invisible. And I had to interject. I am his wife” (Louise, wife).

Her observations at her husband's bedside lead her to believe that the healthcare providers were not talking to one another and therefore, she had to stay as close to him as possible to make sure he would receive appropriate care. The communication gaps resulted in care that did not appear to be focused on Bob's needs, which included dementia, a broken arm and broken hip—the reason he was in the hospital. These gaps in communication also contributed to Louise's belief that she had to be a strong advocate for her husband.

### 4.2 Person‐centred care

Older people and their families wanted the healthcare providers to provide care that recognized the older person as unique. Family members perceived open communication with the older person, them and among team members as evidence that the professionals were “looking at the whole person” (Sandy, daughter) and this decreased their anxiety about whether or not their family member's needs would be addressed. If the older person was receiving care that centred on his or her needs, then family did not need to advocate for the older family member. When communication was evident among the team members and with the older person and the family, the care was perceived as *consistency*. When communication was perceived as lacking or absent, then older people felt *devalued* and their family members perceived a strong need to advocate for their older family member.

#### 4.2.1 Consistency

Family members viewed professionals talking among one another as evidence that they were concerned about providing uniform care that took the unique needs of the older person into consideration. They recognized that it took a team to provide around the clock care for their family member and to do that effectively professionals would need to communicate with one another. When interprofessional team members were talking to one another, it was viewed as evidence that “everybody wanted to make sure they were looking after him properly, and it was a wonderful example of teamwork” (Louise, wife). Another daughter identified that interprofessional communication fostered consistency in the care her father received. “Consistency. I think the team actually works as a team” (Sandy, daughter). Consistency was only possible if the members of the team were talking and listening to one another as well as to the family. If care was perceived as consistent, or if something happened and it was explained, then families were less anxious and were less likely to feel the need to strongly advocate for their older family member. The consistent communication among the interprofessional team was seen as evidence that there was a unit culture of concern for older patients.

Unfortunately, not all of the participants’ experiences were of a culture that exhibited concern for the older person. One family described their experience in which they perceived that their older family member's needs were not considered nor was there communication to them about what was going on with him. Rather, his needs were inconsistently met. One night they got a call from the nursing home where their older family member resided explaining that he had fallen. They spent time in the emergency department with him, yet were never told by professionals at either the nursing home, or the emergency department about what had happened, or the implications of the fall to his health. “I don't know exactly the whole situation. He fell on his face and broke his nose and cut his forehead open.” (Grace, wife). The next day the family attended a meeting with the interprofessional team to discuss his care, a meeting that had been scheduled long before the fall. The fall was not mentioned, nor was his recent weight loss, diabetes and dementia. Rather the conversation focused on the financial implications to the nursing home in providing one‐to‐one care to monitor his wandering. The lack of discussion on his care needs increased the family's anxiety about their family member and made them question the interprofessional teams’ interest in providing care centred on his needs. It also led them to believe they had to be the ones to strongly advocate for their family member's health concerns, particularly because his dementia had advanced to the stage where he was no longer able to advocate for himself.

#### 4.2.2 Devaluing of older people

Many of the family members shared their belief that there was a devaluing of older people within healthcare institutions. This belief was linked to experiences in which their older family member's needs were not communicated among the healthcare team and/or they perceived that the older person did not have their needs met. This inadequate communication was viewed as a reflection of the healthcare system not supporting care of older people. “Don't spend money on a senior, because they're on their way out. Spend money on someone younger. They are more valuable to society” (Ray, son). Families believed that fiscal restraints caused time pressures, which contributed to rushed or diminished communication with older people and their families. This resulted in, older people feeling like “you're not a person. You're a task” (Brent, son). Another wife echoed these sentiments when she suggested that lack of communication contributed to “patients not feeling that they are cared about. They feel like a burden and the families, they feel like they are in the way” (Louise, wife). When healthcare providers failed to communicate about his needs, which necessitated him to be mobilized in an unusual manner, the older person's perception was: “they didn't give a damn about you” (Bob, older person). It would seem that lack of communication was viewed as a lack of concern for the older person.

Families identified that they did expect that sometimes care or communication would be less than perfect. However, if care provided did not take the care needs of the older person into consideration and healthcare providers communicated what had gone wrong, then the family felt reassured that healthcare providers did consider the needs of their older family member overall. Families’ belief that they needed to assume strong advocacy for their older family member to ensure that their needs were considered hinged on whether or not healthcare providers communicated openly when unexpected things happened or if something went wrong.

## 5 DISCUSSION

To our knowledge, this is the first study providing evidence about older people and families perspectives about engaging with interprofessional teams. Findings from this study illuminate and provide depth of knowledge about how older people and their families’ perceived the importance of communication about what was happening in the care of the older person, regardless of the nature of the news. Central to older people and families’ concerns was the need to take into consideration the older person's unique needs in care planning. Communication with older people, their family and with the healthcare team was viewed as crucial in order to provide care that considered the older person's needs. Family members felt the need to strongly advocate for their older family member if their older family member received care that did not meet his or her needs and the reasons for this were not communicated to them. Thus, the findings of this study provide valuable insights into the perspectives of older people and their families, about their communication and care needs.

This study contributes to an emerging body of literature examining family caregiving for older people (Funk, 2010; Jacelon, 2006; Li et al., 2003). Similar to the families in this study, others have identified that families consider it part of their responsibility to be involved in supporting family members as they age (Funk, 2010; O'Connor, Pollitt, Brook, Reiss, & Roth, 1991). Jacelon's (2006) study identified that families were a moderating factor in the hospitalization of older people by acting as an advisor, making decisions when the older patients were unable to do so and supporting (often emotionally) older patients through their visits. Although these studies reported on family involvement in older adults’ healthcare concerns, there was little mention of how interprofessional teams either facilitated or deterred family involvement. This study sheds light on how professionals’ communication about the older person's unique needs with the older person and their family is a significant factor in whether or not families believe they need to take a strong advocate role on behalf of their family member.

Much of the literature about interprofessional collaboration focuses on issues among professionals, rather than how care recipients (not necessarily older people) and their families’ perspectives are incorporated. One exception is a study that examined families’ involvement in intensive care units, which identified that family members provided continuity when communication among interprofessional team members was inadequate (Reeves et al., 2016). Similarly, in this study, families felt it was necessary to step in and advocate for their family member, when they were unable to advocate for themselves and when interprofessional team communication was fractured. Communication and team cohesion can support older people and families’ experiences with interprofessional teams (Jacelon, 2006; Kilpatrick, Jabbour, & Fortin, 2016). Our findings suggest that families who witness and experience interprofessional communication perceive a culture of concern for their older family member. Scholars have illustrated that communication occurs through behaviours, feelings and thoughts about what is occurring (Wright & Leahey, 2007). Thus, being ill or having an ill family member can affect how older people and their families communicate, just as a chaotic work environment can influence how healthcare professionals communicate. In this study, having dementia affected many of the older people's ability to communicate with healthcare professionals, leading to more reliance on their families.

Older people and their families in this study also valued healthcare professionals’ care strategies that took the perspectives of the older person into consideration—often articulated as person‐centred care. Morgan and Yoder's (2012) concept analysis of person‐centred care identified a long history of the use of this concept within health care, despite a lack of consensus on its meaning. They suggested common attributes of person‐centred care as holistic, individualized, respectful and empowering. McCormack (2003) identified that respect for values in providing person‐centred care for older people included learning about how they made sense of what was happening. The findings from this study point to the importance of family members in helping older people (particularly if cognitively impaired) understand what was happening to them, highlighting the need for professionals to collaborate with family members.

Reeves et al.'s (2010) framework identified contextual issues such as the organizational or unit culture as affecting interprofessional team collaboration. Older people and their families in this study described team culture as either positive or negative depending on their experiences with that team. Positive experiences were reported when families were included as part of the older person's team through open communication and when the older person's needs were recognized. Since this study did not include the interprofessional teams’ perspectives we do not know how institutional support may have contributed to the teams’ ability to include family members. More research aimed at understanding how these issues could contribute to concrete strategies for improving interprofessional communication with older people and their families is required.

## 6 IMPLICATIONS

The findings from this study suggest that if interprofessional teams communicate regularly with older people and their families about care, they are more likely to incorporate older peoples’ needs, particularly in the context of cognitive impairment. While the importance of communication seems obvious, this study highlights the pivotal role communication has in how families interpret their need to be a strong advocate for older people and how families view the teams’ interest in meeting their family members’ needs. Nurses have a pivotal role in facilitating communication between various professionals and older people and their families. Moreover, listening to older people and their families about the older person's unique needs and passing this information to the interprofessional team is a key role for nursing. Most importantly, understanding that older people and their families just what to understand what is going on even if it is unknown could encourage nurses and other professionals in communicating regularly. More research into understanding how to enhance effective communication with older people and their families is warranted. Such research could provide guidance to healthcare leaders in planning for the time and infrastructure needed to promote interprofessional collaboration with older adults and their families.

## 7 LIMITATIONS

This study is limited in size, scope and context. Although we endeavoured to sample broadly, all but one of the older participants experienced some type of dementia. The existence of dementia limited older peoples’ ability to reflect on their experiences and, as a result, family members strongly influenced the findings. Moreover, most of the participants described their experiences as either good or bad, rather than somewhere in between. There may, therefore, have been bias in recruitment, families with either strongly positive or negative experiences being more likely to participate. Further research that includes larger number of families, a variety of cultures and in different contexts, such as rural and urban settings, could add to an understanding about how interprofessional teams could best interact with older people and their families.

## 8 CONCLUSIONS

This study's examination of older people and their families’ perspectives of interprofessional teams revealed that families are a necessary and integral part of the care of older people, particularly in the context of impaired cognition. Older people and their families wanted interprofessional teams to recognize their important role as advocates, communicate openly and provide person‐centred care. More research is needed to understand the interprofessional teams’ processes in order to better support collaboration with older people and their families.

## AUTHOR CONTRIBUTIONS

Study conception/design, drafting of manuscript, supervision and administrative/technical/material support: Sherry Dahlke; data collection: Sherry Dahlke and Marnie Colborne; data analysis: Sherry Dahlke, Kim Steil, Rosalie Freund‐Heritage, Marnie Colborne, Susan Labonte and Adrian Wagg; critical revisions for important intellectual content: Sherry Dahlke and Adrian Wagg.

## RESEARCH ETHICS COMMITTEE APPROVAL

Research Ethics Committee approval was obtained from the University of Alberta, Canada, and Alberta Health Services.

## References

[nop2123-bib-0001] Arbaji, A. I. , Maron, D. D. , Yu, Q. , Wnedel, V. I. , Tanner, E. , Boult, C. , … Durso, S. C. (2010). The geriatric floating interdisciplinary transistion team. Journal of the American Geriatrics Society, 58, 364–370. https://doi.org/10.1111/j.1532-5415.2009.02682 20370860 10.1111/j.1532-5415.2009.02682.x

[nop2123-bib-0002] Barrow, M. , McKimm, J. , Gasquoine, S. , & Rowe, D. (2014). Collaborating in healthcare delivery: Exploring conceptual differences at the bedside. Journal of Interprofessional Care, 1356–1820, https://doi.org/10.3109/13561820.2014.955911 10.3109/13561820.2014.955911 25188211

[nop2123-bib-0003] Berglund, H. , Wilhelmson, K. , Blomberg, S. , Duner, A. , Kjellgren, K. , & Hasson, H. (2013). Older people's views of quality of care: A randomized controlled study of continuum of care. Journal of Clinical Nursing, 22, 2934–2944. https://doi.org/10.1111/jocn.12276 23808647 10.1111/jocn.12276

[nop2123-bib-0004] Boult, C. , Green, A. F. , Boult, L. B. , Pacala, J. T. , Snyder, C. , & Leff, B. (2009). Successful models of comprehensive care for older adults with chronic conditions: Evidence for the institute of medicine's retooling for an aging America report. Journal of American Geriatrics Society, 57, 2328–2337. https://doi.org/10.1111/j.1532-5415.2009.02571 10.1111/j.1532-5415.2009.02571.x 20121991

[nop2123-bib-0005] Brandt, B. , Lutfiyya, M. N. , King, J. A. , & Chioreso, C. (2014). A scoping review of interprofessional collaborative practice and education using the lens of the Triple Aim. Journal of Interprofessional Care, 28(5), 1–8. https://doi.org/10.3109/13561820.2014.906391 10.3109/13561820.2014.906391 PMC4162503 24702046

[nop2123-bib-0006] Canada, H. (2007). Primary health care transition fund: Summary of intitiatives. Ottawa: Health Canada.

[nop2123-bib-0007] Dahlke, S. , Meherali, S. , Chambers, T. , Freund‐Heritage, R. , Steile, K. , & Wagg, A. (2017). The care of older adults experiencing cognitive challenges: How interprofessional teams collaborate. Canadian Journal on Aging, 36(4), 485–500. https://doi.org/10.1017/S0714980817000368 28920561 10.1017/S0714980817000368

[nop2123-bib-0008] Eloranta, S. , Routasalo, P. , & Arve, S. (2008). Person resources supporting living at home as described by older home care clients. International Journal of Nursing Practice, 14, 308–314. https://doi.org/10.111/j.1440-172X.2008.00698.x 18715393 10.1111/j.1440-172X.2008.00698.x

[nop2123-bib-0009] Essen, C. , Freshwater, D. , & Cahill, J. (2015). Towards an understanding of the dynamic sociomaterial embodiment of interprofessional collaboration. Nursing Inquiry, 22, 1–11.https://doi.org/10.1111/nin.12093 25665092 10.1111/nin.12093

[nop2123-bib-0010] Fedarko, N. S. (2011). The biology of aging and frailty. Clinical Geriatric Medicine, 27, 27–37. https://doi.org/10.1016/j/cger.2010.08.006 10.1016/j.cger.2010.08.006 PMC3052959 21093720

[nop2123-bib-0011] Finn, R. , Learmonth, M. , & Reedy, P. (2010). Some unintended effects of teamwork in healthcare. Social Science and Medicine, 70, 1148–1154. https://doi.org/10.1016/j.cpcscormed.2009.12.025 20137845 10.1016/j.socscimed.2009.12.025

[nop2123-bib-0012] Fox, A. , & Reeves, S. (2015). Interprofessional collaborative patient‐centered care: A critical exploration of two related discourses. Journal of Interprofessional Care, 29(2), 113–118. https://doi.org/10.3109/13561820.2014.954284 25180630 10.3109/13561820.2014.954284

[nop2123-bib-0013] Funk, L. M. (2010). Prioritizing parental autonomy: Adult children's accounts of feeling responsible and supporting aging parents. Journal of Aging Studies, 24, 57–64, https://doi.org/10.1016/j.jaging2008.03.003

[nop2123-bib-0014] Grove, S. K. , Gray, J. R. , & Burns, N. (2015). Understanding nursing research: Building and evidence‐based practice (Edn). St. Louis: Elsevier.

[nop2123-bib-0015] Hartgerink, J. M. , Cramm, J. M. , Bakker, T. J. E. M. , van Eijsden, A. M. , Mackenbach, J. P. , & Nieboer, A. P. (2014). The importance of multidisciplinary teamwork and team climate for relational coordination among teams delivering care to older patients. Journal of Advanced Nursing, 70(4), 791–799. https://doi.org/10.1111/jan.12233 23980594 10.1111/jan.12233 PMC4282281

[nop2123-bib-0016] Hochhalter, A. K. , Song, J. , Rush, J. , Sklar, L. , & Stevens, A. (2010). Making the most of your healthcare intervention for older adults with multiple chronic illnesses. Patient Education and Counseling, 81, 207–213. https://doi.org/10.1016/j.per.2010.01.018 20223617 10.1016/j.pec.2010.01.018

[nop2123-bib-0017] Holroyd‐Leduc, J. , Khandwala, F. , & Sink, K. M. (2010). How can delirium best be prevented and managed in older patients in hospital? Canadian Medical Association Journal, 182(5), 465–470. https://doi.org/10.1503/cmaj.080519 19687107 10.1503/cmaj.080519 PMC2842841

[nop2123-bib-0018] Hsieh, H. , & Shannon, S. E. (2005). Three approaches to qualitative content analysis. Qualitative Health Research, 15(9), 1277–1288. https://doi.org/10.1177/1049732305276687 16204405 10.1177/1049732305276687

[nop2123-bib-0019] Jacelon, C. S. (2006). Directive and supportive behaviors used by families of hospitalized older adults to affect the process of hospitalization. Journal of Family Nursing, 12(3), 234–250. https://doi.org/10.1177/107/4840706290264 16837693 10.1177/1074840706290264

[nop2123-bib-0020] Jones, A. , & Jones, D. (2011). Improving teamwork, trust and safety: An ethnographic study of an interprofessional initiative. Journal of Interprofessional Care, 23(3), 175–181. https://doi.org/10.3109/13561820.2010.520248 10.3109/13561820.2010.520248 21043559

[nop2123-bib-0021] Kilpatrick, K. , Jabbour, M. , & Fortin, C. (2016). Processes in healthcare teams that include nurse practitioners: What do patients and families perceive to be effective? Journal of Clinical Nursing, 25, 619–630. https://doi.org/10.1111/jocn.13085 26875841 10.1111/jocn.13085

[nop2123-bib-0022] Lamb, B. W. , Jalil, R. T. , Shah, S. , Brown, K. , Allchorne, P. , Vincent, C. , … Sevdalis, N. (2014). Cancer patients’ perspectives on multidisciplinary team working: An exploratory focus group study. Urologic Nursing, 34(2), 83–91. https://doi.org/10.7257/1053-816X.2014.2.83 24919246

[nop2123-bib-0023] Legare, F. , Stacey, D. , Briere, N. , Robitaille, H. , Lord, M. C. , & Desroches, S. (2014). An interprofessional approach to shared decision making: An exploratory case study with family caregivers of one IP home care team. BioMed Central Geriatrics, 14, 83–90. 24985335 10.1186/1471-2318-14-83 PMC4105553

[nop2123-bib-0024] Lemieux‐Charles, L. , & McGuire, W. L. (2006). What do we know about healthcare team effectiveness? A review of the literature. Medical Care Research and Review, 63(3), 263–300. https://doi.org/10.1177/1077558706287003 16651394 10.1177/1077558706287003

[nop2123-bib-0025] Li, H. , Melnyk, B. M. , McCann, R. , Chatcheydang, J. , Koulouglioti, C. , Nichols, L. W. , … Ghassemi, A. (2003). Creating avenues for relative empowerment (CARE): A pilot test of an intervention to improve outcomes of hospitalized elders and family caregivers. Research in Nursing & Health, 26, 284–299. https://doi.org/10.1002/nur.10091 12884417 10.1002/nur.10091

[nop2123-bib-0026] Manser, T. (2009). Teamwork and patient safety in dynamic domains of healthcare: A review of the literature. Anesthesiology Scandinavia, 53, 143–151. https://doi.org/10.1111/j.1399-6576.2008.01717.x 10.1111/j.1399-6576.2008.01717.x 19032571

[nop2123-bib-0027] McCormack, B. (2003). A conceptual framework for person‐centered practice with older people. International Journal of Nursing Practice, 9, 202–209. https://doi.org/10.1046/j.1440-172X.2003.00423.x 12801252 10.1046/j.1440-172x.2003.00423.x

[nop2123-bib-0028] Mickan, S. M. , & Rodger, S. A. (2005). Effective health care teams: A model of six characteristics developed from shared perceptions. Journal of Interprofessional Care, 19(4), 358–370. https://doi.org/10.1080/13561820500165142 16076597 10.1080/13561820500165142

[nop2123-bib-0029] Morey, J. C. , Simon, R. , Jay, G. D. , Wears, R. L. , Salisbury, M. , Dukes, K. A. , & Berns, S. D. (2002). Error reduction and performance improvement in the emergency department through formal teamwork training: Evaluation results of the MedTeams Project. Health Services Research, 37(6), 1553–1581. https://doi.org/10.1111/1475-6773.01104 12546286 10.1111/1475-6773.01104 PMC1464040

[nop2123-bib-0030] Morgan, S. , & Yoder, L. H. (2012). A concept analysis of person‐centered care. Journal of Holistic Nursing, 30(1), 6–15. https://doi.org/10.1177/0898010111412189 21772048 10.1177/0898010111412189

[nop2123-bib-0031] O'Connor, D. W. , Pollitt, P. A. , Brook, C. P. B. , Reiss, B. B. , & Roth, M. (1991). Does early intervention reduce the number of elderly people with dementia admitted to institutions for long term care? British Medical Journal, 302, 871–875. https://doi.org/10.1136/bmj.302.6781.871 1902752 10.1136/bmj.302.6781.871 PMC1669209

[nop2123-bib-0032] Paradis, E. , Leslie, M. , Puntillo, K. , Gropper, M. , Aboumatar, H. J. , Kitto, S. , & Reeves, S. (2014). Delivering interprofessional care in intensive care: A scoping review of ethnographic studies. American Journal of Critical Care, 23(3), 230–238. https://doi.org/10.4037/ajcc2014155 24786811 10.4037/ajcc2014155

[nop2123-bib-0033] Reeves, S. (2012). The rise and rise of interprofessional competence. Journal of Interprofessional Care, 26, 253–255. https://doi.org/10.3109/13561820.695542 22676141 10.3109/13561820.2012.695542

[nop2123-bib-0034] Reeves, S. , Lewin, S. , Espin, S. , & Zwarenstein, M. (2010). Interprofessional teamwork for health and social care: Promoting partnership for health. Chichester, UK: Wiley‐Blakewell https://doi.org/10.1002/9781444325027

[nop2123-bib-0035] Reeves, S. , McMillan, S. E. , Kachan, N. , Paradis, E. , Leslie, M. , & Kitto, S. (2016). Interprofessional collaboration and family member involvement in intensive care units: Emerging themes from a multi‐sited ethnography. Journal of Interprofessional Care, 29(3), 230–237. https://doi.org/10.3109/13561820.2014.955914 10.3109/13561820.2014.955914 25238573

[nop2123-bib-0036] Rowlands, S. , & Callen, J. (2013). A qualitative analysis of communication between members of a hospital‐based multidisciplinary lung cancer team. European Journal of Cancer Care, 22, 20–31. https://doi.org/10.1111/ecc.12004 22966875 10.1111/ecc.12004

[nop2123-bib-0037] Sandelowski, M. (2000). Whatever happened to Qualitative description? Research in Nursing and Health, 23, 334–340. https://doi.org/10.1002/(ISSN)1098-240X 10940958 10.1002/1098-240x(200008)23:4<334::aid-nur9>3.0.co;2-g

[nop2123-bib-0038] Sandelowski, M. (2010). What's in a name? Qualititative description revisited. Research in Nursing and Health, 33, 77–84. https://doi.org/10.1002/nurs.20362 20014004 10.1002/nur.20362

[nop2123-bib-0039] Schmitt, M. H. (2001). Collaboration improves the quality of care: Methodological challenges and evidence from US healthcare research. Journal of Interprofessional Care, 15(1), 47–66. https://doi.org/10.1080/1356182002002873 11705070 10.1080/13561820020022873

[nop2123-bib-0040] Schmitt, M. H. (2006). Interprofessional approaches to creating safe quality healthcare. Journal of Interprofessional Care, 20(5), 455–457. https://doi.org/10.1080/13561820600959303

[nop2123-bib-0041] Smith‐Carrier, T. , & Neysmith, S. (2014). Analyzing the interprofessional working of a home‐based primary care team. Canadian Journal on Aging, 33(3), 271–284. https://doi.org/10.1017/S0714981400021 26261888 10.1017/S071498081400021X

[nop2123-bib-0042] Wolf, M. S. , Gazmararian, J. A. , & Baker, D. W. (2005). Health literacy and functional health status among older adults. Archives Internal Medicine, 165, 1946–1952. https://doi.org/10.1001/archinte.165.17.1946 10.1001/archinte.165.17.1946 16186463

[nop2123-bib-0043] World Health Organization (WHO) (2010). Framework for action on interprofessional education and collaborative practice. Genevia: WHO. 21174039

[nop2123-bib-0044] World Health Organization (WHO) . (2015). The epidemiology and impact of dementia: Current state and future trends. Genevia: WHO Available from www.who.int/mental_health/neurology/dementia/dementia_thematicbrief_epidemiology.pdf [last accessed November 2015]

[nop2123-bib-0045] World Health Organization (WHO) (2016). World alzheimer report 2016: Improving healthcare for people living with dementia. London: Alzheimer's disease International.

[nop2123-bib-0046] Wright, L. M. , & Leahey, M. (2007). Nurses and families: A guide to family assessment and intervention. Philadelphia: F.A. Davis Company.

